# The increasing burden and complexity of multi-morbidity and polypharmacy in geriatric HIV patients: a cross sectional study of people aged 65 – 74 years and more than 75 years

**DOI:** 10.1186/s12877-018-0789-0

**Published:** 2018-04-20

**Authors:** G. Guaraldi, A. Malagoli, A. Calcagno, C. Mussi, B. M. Celesia, F. Carli, S. Piconi, G. V. De Socio, A. M. Cattelan, G. Orofino, A. Riva, E. Focà, S. Nozza, G. Di Perri

**Affiliations:** 1 Infectious Disease Clinic, Department of Medical and Surgical Sciences for Children & Adults, University of Modena and Reggio Emilia, Modena, Italy; 2 Unit of Infectious Diseases, Department of Medical Sciences, University of Turin, Turin, Italy; 3 Centre of Gerontological Evaluation and Research, University of Modena and Reggio Emilia, Modena, Italy; 4 Department of Clinical and Molecular Biomedicine, Division of Infectious Diseases, University of Catania, ARNAS Garibaldi, Catania, Italy; 5 First Division of Infectious Diseases Unit, University of Milan, Ospedale L. Sacco, Milan, Italy; 6 Department of Infectious Diseases, Azienda Ospedaliero-Universitaria di Perugia, Perugia, Italy; 7 Unit of Infectious Diseases, Department of Internal Medicine, Azienda Ospedaliera-Universitaria di Padova, Padua, Italy; 8 Unit of Infectious Diseases, Division A, Ospedale Amedeo di Savoia, ASLTO2, Turin, Italy; 9 Third Division of Infectious Diseases, University of Milan, Ospedale L. Sacco, Milan, Italy; 10 Unit of Infectious and Tropical Diseases, University of Brescia, Brescia, Italy; 11 Department of Infectious Diseases, San Raffaele Scientific Institute, Milan, Italy

**Keywords:** Geriatric HIV-infected population, Multi-morbidity, Polypharmacy

## Abstract

**Background:**

Geriatric Patients Living with HIV/AIDS (GEPPO) is a new prospective observational multicentre cohort consisting of all the HIV-positive geriatric patients being treated at 10 clinics in Italy, and HIV-negative controls attending a single geriatric clinic.

The aim of this analysis of the GEPPO cohort was to compare prevalence and risk factors of individual non-communicable diseases (NCD), multi-morbidity (MM) and polypharmacy (PP) amongst HIV positive and HIV negative controls at enrolment into the GEPPO cohort.

**Methods:**

This cross-sectional study was conducted between June 2015 and May 2016. The duration of HIV infection was subdivided into three intervals: < 10, 10–20 and > 20 years. The NCD diagnoses were based on guidelines defined criteria, including cardiovascular disease, hypertension, type 2 diabetes, chronic kidney disease, dyslipidaemia, chronic obstructive pulmonary disease. MM was classified as the presence of two or more co-morbidities. The medications prescribed for the treatment of comorbidities were collected in both HIV positive and HIV negative group from patient files and were categorized using the Anatomical Therapeutic Chemical (ATC) classification. PP was defined as the presence of five or more drug components other than anti-retroviral agents.

**Results:**

The study involved a total of 1573 patient: 1258 HIV positive and 315 HIV negative). The prevalence of individual comorbidities was similar in the two groups with the exception of dyslipidaemia, which was more frequent in the HIV-positive patients (*p* <  0.01). When the HIV-positive group was stratified based on the duration of HIV infection, most of the co-morbidities were significantly more frequent than in control patients, except for hypertension and cardiovascular disease, while COPD was more prevalent in the control group. MM and PP were both more prevalent in the HIV-positive group, respectively 64% and 37%.

**Conclusions:**

MM and PP burden in geriatric HIV positive patients are related to longer duration of HIV-infection rather than older age per se.

## Background

Aging populations are about to become the next global challenge for global public health. Advances in medicine and socio-economic development have substantially reduced morbidity and mortality due to infectious conditions and, to some extent, non-communicable diseases (NCD) [1]. Moreover, the longer survival of people with chronic conditions explains the increasing proportion of people living with NCDs. The co-existence of two or more NCDs is usually defined as multi-morbidity (MM) [2, 3]. Empirical studies based on surveys and general practice records show that MM is highly prevalent among older adults [4], and is associated with more medication prescriptions (polypharmacy, PP), the greater use of healthcare services, greater disability and mortality, and a poorer health-related quality of life [5–7]. The demographic shift has led gerontologists to recognise the different conditions that people experience during the years known as geriatric age, above 65 years. Furthermore, it has brought with it the widely used sub-grouping into the youngest-old (65–74 years), the old (75–84 years), and the oldest-old (≥ 85 years) [8].

In the relatively new context of global aging, Human Immunodeficiency Virus (HIV) infection is less an exception than a paradigm. The increasing age of people living with HIV (PLWH) is the net result of increased survival due to effective antiretroviral therapy (ART) and older age at the time the infection is acquired [9]. A few studies have assessed the clinical presentation of aging HIV patients, particularly the proportion of age-related NCD affecting those aged > 50 years [10–14].

Two European cohorts (POPPY and AgeHIV) identified well-matched HIV-negative subjects. This allowed to study the impact of HIV specific risk factors such as ART exposure and toxicity, immune dysfunction or dysregulation, and chronic immune activation and inflammation [14–19]. Unfortunately, median age of these cohorts are below 50 years and none of these studies have a significant proportion of subjects of appropriately defined geriatric age.

The clinical characteristics of more than 400 patients aged > 75 years in a large French database were described at the 2016 Conference on Retroviruses and Opportunistic Infection (CROI, Boston, 22–25 February 2016). However, they acquired HIV infection at a late age (the median age at the time of starting ART being 64.5 years, range 60–70) [20]. These data have not been published so far. Therefore, the clinical presentation and aging trajectory of geriatric patients aging with HIV infection is still unknown.

Geriatric Patients Living with HIV/AIDS (GEPPO) is a new prospective observational multicentre cohort including consecutive HIV-positive geriatric patients in care at 10 HIV clinics in Northern Central and Southern Italy, and HIV-negative controls attending a single geriatric clinic.

The overall aims of the GEPPO study are to determine the health status of HIV-positive patients aged ≥65 years and its changes over time. A further aim is to investigate the extent to which the geriatric care model applies to HIV positive patients. Finally, it is intended to identify the contemporary morbidity, mortality and disability factors affecting healthy life expectancy of geriatric HIV positive patients.

In this analysis we compared prevalence and risk factors of individual non-communicable diseases, multi-morbidity and polypharmacy amongst HIV positive and HIV negative controls at enrolment into the GEPPO cohort. Cross-sectional comparison was stratified by age groups, namely: youngest old (65–75) and old (≥75 years).

## Methods

This is a cross-sectional analysis of HIV positive and HIV negative geriatric patients at the time of GEPPO cohort entry between June 2015 and May 2016. The patients were recruited at the time of routine follow up visit at ten HIV clinics in Northern, Central and Southern Italy with a geographical spread of 1000 Km, and were stratified into two groups: the “youngest old” (65–74 years) and the “old” (≥ 75 years). The inclusion criteria were age of ≥65 years, treatment with ART for at least six months and signed informed consent.

The HIV-negative subjects were selected from those attending a single geriatric clinic located in the same geographical area as the coordinating site (Modena). This centre offers, general practitioners support in screening NCDs in geriatric patients. The only inclusion criterion was age ≥ 65 years. Given the easy access and free of charge of any diagnostic procedure in geriatric patients, this cohort is representative of the general Italian population.

### Ethics

Institutional review Board (IRB) approval was obtained from the Research Ethics Committee of each centre participating in the GEPPO cohort study. Both HIV positive and HIV negative participants gave their written informed consent, at the time of their initial visit.

### Covariates

The demographic covariates and clinical outcomes of the HIV positive and HIV negative subjects were characterised and compared. They included: age, gender, BMI, smoking status. Ex- and never-smokers were grouped together and compared to current smokers. The variables considered in HIV positive patients included: current and nadir CD4 cell counts, CD4/CD8 ratio, plasma HIV RNA viral load (VL). The duration of HIV infection was calculated as the time between diagnosis and the last visit, and was stratified into < 10, 10–20 and > 20 years. The duration of ART was calculated as the time between the start of ART and the last visit.

### Outcomes

The NCDs diagnoses were based on guidelines defined criteria [21]. The cardiovascular disease (CVD) category consisted of diagnoses of myocardial infarction, coronary artery disease, peripheral vascular disease, stroke and angina pectoris, as well as coronary artery bypass grafting and angioplasty, based on records in patient files. Hypertension (HTN) was defined as two consecutive measurements of blood pressure > 140/90 mmHg or use of antihypertensive drugs. Type 2 diabetes mellitus (T2DM) was defined as fasting serum glucose levels ≥126 mg/dL or use of antidiabetic drugs. Chronic kidney disease (CKD) was confirmed at an estimated glomerular filtration rate (eGFR) of < 60 mL/min calculated using Chronic Kidney Disease Epidemiology Collaboration (CKD-Epi) equation mL/min/1.73 m2. Dyslipidaemia (DLM) was defined in patients with fasting total cholesterol levels > 200 mg/dL or triglyceride levels of > 150 mg/dL or the current use of statins. Diagnosis of HTN, CKD and DLM were confirmed in two consecutive measurements. Chronic obstructive pulmonary disease (COPD) was defined based on pulmonary function tests (spirometry, diffusion capacity of carbon monoxide [DLCO]) demonstrating FEV_1_/forced vital capacity (FVC) ratios < 70%. MM was defined as the presence of two or more NCDs [2, 3].

The medications prescribed for the treatment of NCDs were collected from patient files in both HIV positive and HIV negative groups and were categorized using the Anatomical Therapeutic Chemical (ATC) classification in which the drugs are divided into different groups based on therapeutic indication [22]. The analysis considered the prevalence of the six most frequently prescribed classes other than ART with particular regards of cardiovascular active agents including statins, beta-blocker, ACE-inhibitors, anti-hypertensives and acetyl-salicylic acid (ASA) and psychoactive agents including benzodiazepines (BDZ).

Polypharmacy was defined as the presence of five or more drug components other than ART. The decision not to include ART was due to the need to compare HIV-positive and HIV-negative subjects.

### Statistical analysis

In the participating Centres the study size of the HIV-positive patients was represented by the whole of HIV infected patients meeting inclusion criteria, who presented at routine medical visits in the enrolment period (year 2015).

Per protocol the two groups were matched for age (±4 years) within male and female groups through a 4:1 ratio, using random selection. A reduced number of HIV negative people were chosen with a view to the large sample size of HIV patients.

Missing data on outcomes were indicated in the tables as different denominators for parentage values.

The between-group comparisons were made using the χ^2^ test for categorical variables, and the t test or Mann-Whitney U-test for the normally and non-normally distributed continuous variables respectively.

The probability of MM and PP at each age was compared in the HIV-negative controls and the HIV-positive patients stratified as of the duration of HIV infection (< 10, 10–20 and > 20 years). These times were chosen for two main reasons. Firstly they paralleled the tertile distribution of this variable. Secondly, they identified the subsets of subjects aging since the pre-ART, and the early and the late-ART periods.

Multivariable logistic regression models were built to predict MM and PP including the following as covariates: age categories, gender, BMI, current smoke and duration of HIV infection, using HIV negative as reference.

A second model was restricted to HIV patients including HIV related variables such as: current CD4, CD4 Nadir, CD4/CD8 ratio, HIV-1 VL undetectability (< 40 copies/mL), and residual of ART duration after adjustment for HIV infection duration.

Residual ART duration was calculated through univariate linear regression between ART exposure and duration of HIV infection. This was performed to avoid co-linearity between these two variables.

Statistical analyses were performed using the “R” Software, version 3·2.

## Results

The study involved a total of 1573 patient (1258 HIV positive and 315 HIV negative). The HIV-positive patients aged 65–74 and ≥ 75 respectively represented 3.8% and 0.5% of the HIV-positive populations at the GEPPO recruiting sites.

With regards to demographic and anthropometric variables, mean age was 72 (SD = 4.27) years for men and 71 (SD = 3.94) years for women. HIV positive individuals were thinner and more frequent smokers. In the group of individuals above the age of 75 HIV negative had the same prevalence of smoke habits as HIV-positive ones (10.84% vs 15.23%, *p* = 0.39) (Table 1).

**Table 1:** Demographic, anthropometric and HIV variables in the GEPPO cohort

	Total *n* = 1573	HIV-negative vs HIV-positive			HIV-negative			HIV-positive		
		HIV- (*n* = 315, 20·03%)	HIV+ (*n* = 1258, 79·97%)	*p*	65–74 years (*n* = 224, 71·11%)	≥ 75 years (*n* = 91, 28·89%)	*p*	65–74 years (*n* = 965, 76·71%)	≥ 75 years (*n* = 293, 23·29%)	*p*
Variable	Mean (SD) [sample size]	Mean (SD/%) [sample size]			Mean (SD/%) [sample size]			Mean (SD/%) [sample size]		
Gender (F)	271 (17.23%) [1573]	66 (20.95%) [315]	205 (16.3%) [1258]	0.06 *	42 (18.75%) [224]	24 (26.37%) [91]	0·18 *	155 (16.06%) [965]	50 (15.06%) [293]	0.75 *
Female Age	71.23 (3.94) [271]	71.55 (3.42) [66]	71.15 (4.05) [205]	0.12 ***	69.83 (2.48) [182]	77.46 (2.08) [67]	< 0.01***	69.46 (2.52) [810]	77.5 (2.0) [243]	< 0.01***
Male Age	71.71 (4.27) [1302]	72.61 (4.37) [249]	71.42 (4.21) [1053]	0.06 ***	70.05 (2.75) [42]	75.61 (0.49) [25]	< 0.01***	69.35 (2.48) [155]	77.17 (1.) [50]	< 0.01***
BMI	26.55 (8.59) [1224]	28.65 (4.18) [301]	25.87 (9.5) [923]	< 0.01 ***	28.71 (4.04) [8]	28.52 (4.54) [6]	0.65 ***	26.15 (10.66) [264]	24.98 (3.95) [71]	0.04 ***
Current smoker	313 (23.29%) [1344]	42 (14.24%) [295]	271 (25.83%) [1049]	< 0.01*	33 (15.57%) [212]	9 (10.84%) [83]	0.39 *	234 (29.03%) [806]	37 (15.23%) [243]	< 0.01*
HIV duration (years)	NA	NA	17.17 (7.65) [1240]	NA	NA	NA	NA	17.24 (7.76) [949]	16.92 (7.3) [291]	0·64 **
< 10 years	NA	NA	263 (21.23%) [1240]	NA	NA	NA	NA	200 (21·07%) [949]	63 (21.72%) [291]	0·9*
10–20 years	NA	NA	561 (45.28%) [1240]	NA	NA	NA	NA	433 (45.63%) [949]	128 (44.14%) [291]	
> 20 years	NA	NA	415 (33.49%) [1240]	NA	NA	NA	NA	316 (33.3%) [949]	99 (34.14%) [291]	
CD4 nadir	NA	NA	197.5 (84–310) [1240]	NA	NA	NA	NA	200 (89.75–308) [949]	191 (74.75–320) [291]	0·61 **
Current CD4	NA	NA	644.58 (1240) [1258]	NA	NA	NA	NA	651.23 (290.95) [949]	622.55 (282) [291]	0·18 **
CD4/CD8	NA	NA	0.97 (1.45) [1240]	NA	NA	NA	NA	0.92 (0.8) [949]	1.13 (2.63) [291]	0·28 ***
Viral load ≤40	NA	NA	1044 (94.31%) [1107]	NA	NA	NA	NA	812 (94.97%) [855]	232 (92.06%) [252]	0.11 *
Viral load undetectable	NA	NA	925 (86.53%) [1068]	NA	NA	NA	NA	712 (86.72%) [821]	213 (85.89%) [248]	0.82 *
HBV co-infection	NAv	NAv	103 (9.83%) [1048]	NAv	NAv	NAv	NAv	84 (10.51%) [799]	19 (7.63%) [249]	0·23*
HCV co-infection	NAv	NAv	141 (12.57%) [1121]	NAv	NAv	NAv	NAv	113 (13·11%) [862]	28 (10·77%) [259]	0·12 *
Age at HIV diagnosis	NA	NA	54·03 (8.83) [1239]	NA	NA	NA	NA	52·11 (8.28) [949]	60·3 (7.6) [290]	< 0·01***
Triple/M ART	NA	NA	390 (31.91%) [1222]	NA	NA	NA	NA	312 (31.01%) [1006]	78 (36.11%) [216]	0·17 *
Mono/dual ART	NA	NA	832 (68.09%) [1221]	NA	NA	NA	NA	694 (68.99%) [1006]	138 (63.89%) [215]	

Abbreviations: *ART*: AntiRetroviral Therapy; *BMI*: Body Mass Index; *HIV*: Human Immunodeficiency Virus; *NA*: not applicable. *NAv*: not available;

*p* value legends: * X2 test; ** Wilcoxon; *** t test

With regards to HIV variables mean HIV duration of 17 years. However, 33% of them had HIV exposure for more than 20 years, representing people aging with HIV from the pre-ART era. The age at HIV diagnosis was significantly higher (60.3 ± 7.6 vs 52.1 ± 8.2) in “old” individuals compared to “youngest old” HIV people (*p* <  0.01). HIV positive patients had well recovered immune status, as assessed with a mean CD4/CD8 equal to 0.97 and reached HIV-RNA viral load below 40 copies/mL in 94%. Hepatitis C and B co-infection were 13% and 10% respectively (Table 1).

NCDs, MM and PP prevalence increased with age categories with the exception of T2DM and dyslipidaemia in HIV negative patients only (Table 2).

**Table 2:** NCDs prevalence comparing “youngest old” and “old” individuals

	65–74 Years Old			≥ 75 Years Old		
	HIV- (*n* = 224, 18.84%)	HIV+ (*n* = 965, 81.16%)	*p*	HIV- (*n* = 91, 23.7%)	HIV+ (*n* = 293, 76.3%)	*p*
Variable	Frequency [sample size]			Frequency [sample size]		
HTN	149 (66.52%) [224]	396 (60.83%) [652]	0.15	61 (67.03%) [91]	155 (71.76%) [216]	0.49
T2DM	50 (22.32%) [224]	176 (27.54%) [629]	0.15	14 (15.38%) [91]	65 (31.25%) [208]	< 0.01
CVD	41 (18.3%) [224]	106 (16.88%) [628]	0.70	28 (30.77%) [91]	58 (29.15%) [199]	0.88
CKD	5 (5%) [100]	115 (17.06%) [674]	< 0.01	4 (10%) [40]	56 (25.93%) [216]	0.04
COPD	20 (9.13%) [219]	41 (6.61%) [620]	0.28	17 (18.89%) [90]	19 (9.79%) [194]	0.05
DLM	59 (57.84%) [102]	462 (70%) [630]	0.02	20 (50%) [40]	156 (74.64%) [209]	< 0.01
MM	57 (57.58%) [99]	371 (61.32%) [605]	0.55	25 (62.5%) [40]	139 (73.94%) [188]	0.20
PP	44 (19.64%) [224]	169 (35.28%) [479]	< 0.01	32 (35.16%) [91]	73 (42.94%) [170]	0.27

Abbreviations: *HTN*: Hypertension; *T2DM*: type 2 diabetes mellitus; *CVD*: Cardiovascular Disease; *CKD*: Chronic Kidney Disease; *COPD*: Chronic Obstructive Pulmonary Disease; *DLM*: Dyslipidaemia; *MM*: Multimorbidity; PP - Polypharmacy

The overall prevalence of MM and PP respectively amounted to 64% and 37% in HIV-positive patients, and 59% and 24% in controls.

In “youngest old” group CKD, DLM and PP only were more prevalent in HIV positive patients; the same was true in the “old” group for T2DM, DLM and CKD (Table 2).

**Fig. 1:**
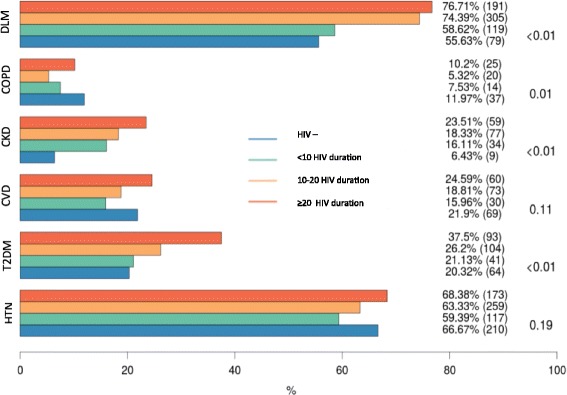
Prevalence of NCDs in the cohort as a whole, and in three HIV duration groups (< 10, 10–20 and > 20 years). NCDs prevalence (%) and absolute numbers (n) are indicated for HIV negative and HIV positive with different HIV duration groups. Indicated *p*-value refers to HIV-positive versus HIV-negative comparison. Abbreviations DLM: Dyslipidaemia; COPD: Chronic Obstructive Pulmonary Disease; CKD: Chronic Kidney Disease; CVD: Cardiovascular Disease; T2DM: Type 2 Diabetes mellitus.HTN: Hypertension

When the HIV-positive group was stratified by duration of HIV infection, individual comorbidities were significantly more frequent in the HIV-positive subgroups with HIV exposure> 10 years when compared to HIV negative, except for HTN and CVD. The prevalence of COPD was higher in the controls (fig. 1).

**Fig. 2:**
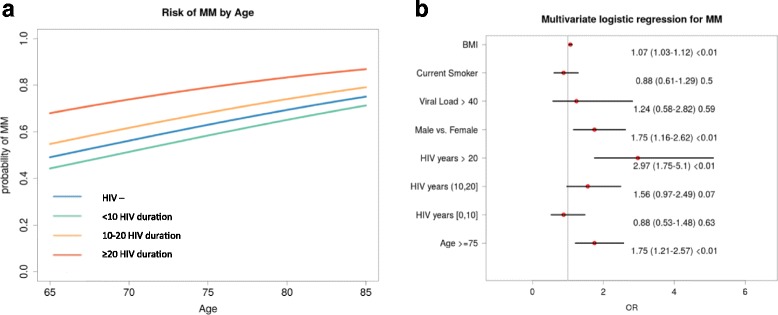
**a** Probability of MM per year above the age of 65. The HIV positive patients are stratified by duration of HIV infection (< 10, 10–20 and > 20 years). **b** Multivariable logistic regression models to detect the independent predictors of MM. Abbreviations – MM: Multimorbidity

Probability of MM was higher in HIV positive patients aging with HIV for more than 10 years when compared to HIV negative controls (fig. 2a). Independent predictors for MM were age > 75 years, higher BMI, male gender and HIV duration above 20 years, all *p* < 0.01 (fig. 2b). A second model restricted to HIV patients only (data not presented in fig. 2), while confirming the same predictors of the previous model, failed to identify any association between traditional HIV variables and MM. In particular: current CD4/CD8 OR = 1.53 (95% CI:0.97–2.43, *p* = 0.07), CD4 nadir OR = 1 (95% CI:1–1, *p* = 0.83), HIV RNA undetectability OR = 1.37 (95% CI:0.65–3.04, *p* = 0.42) and residual ARV exposure duration OR = 0.97 (95% CI:0.9–1.05, *p* = 0.45).

**Fig. 3:**
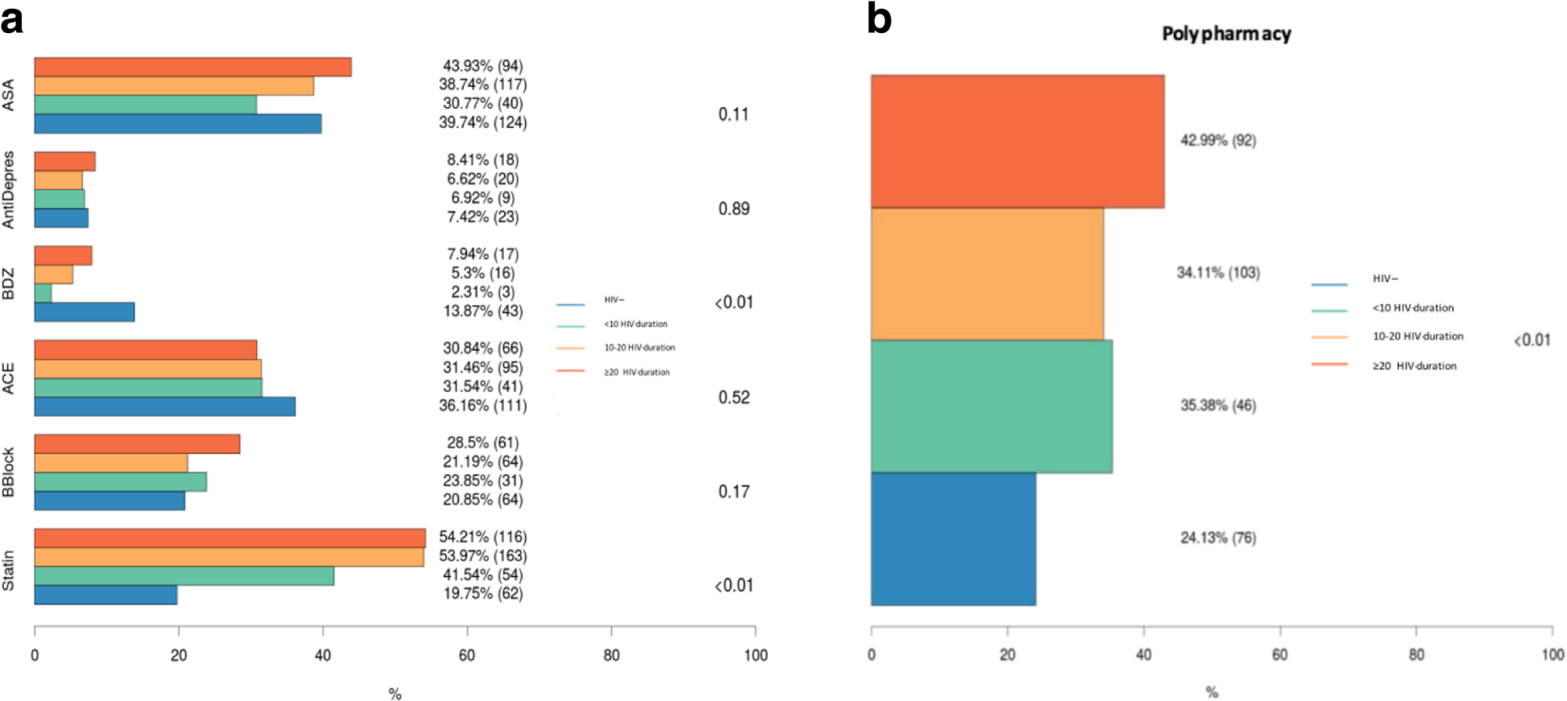
**a** Prevalence of the six drug classes most frequently prescribed for the treatment of NCdD and **b** prevalence of PP in HIV negative and HIV positive stratified by duration of HIV infection (< 10, 10–20 and > 20 years). Drug classes prevalence (%) and absolute numbers (n) are indicated for HIV negative and HIV positive with different HIV duration groups. Indicated p-value refers to HIV-positive versus HIV-negative comparison. Abbreviations – ASA: acetylsalicylic acid; AntiDepres: antidepressants; BZD: benzodiazepines; ACE; Angiotensin Converting Enzyme inhibitors; BBlock: beta-adrenergic blocking agents; Statine: statins

We examined in the GEPPO cohort the six most frequently prescribed drug classes for the treatment of NCDs. There was no difference in the prescription of and antidepressants or acetylsalicylic acid (ASA), ace-inhibitors (ACE) and beta-blockers, commonly used in primary or secondary cardiovascular disease prevention. A higher prescription of benzodiazepines (BDZ), used as sleep inducers, was present in HIV negative people (p < 0.01), while a higher prescription of statins for dyslipidaemia in HIV positive patients was observed (p < 0.01) (fig. 3a). PP was higher in HIV positives, irrespective of duration of HIV-infection (Fig. 3b).

**Fig. 4:**
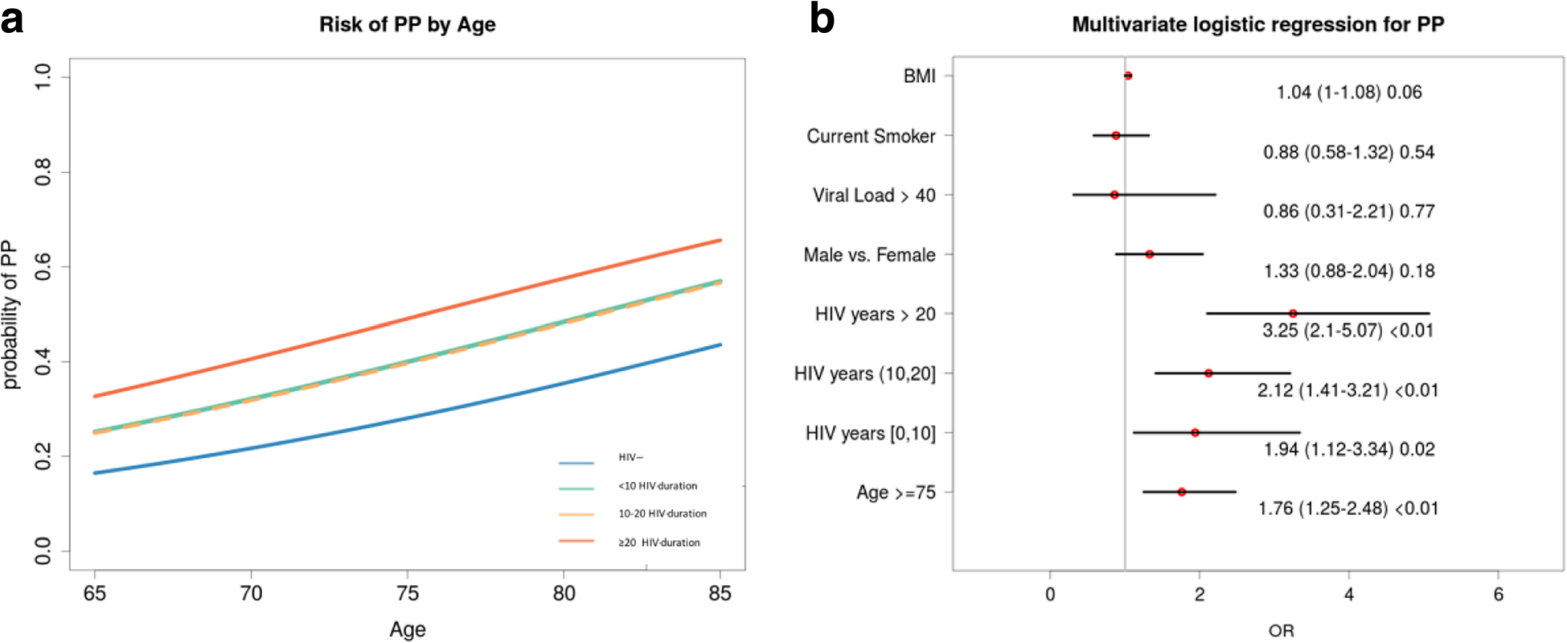
**a** Probability of PP per year above the age of 65. The HIV positive patients are stratified by duration of HIV infection (< 10, 10–20 and > 20 years). **b** Multivariable logistic regression models to detect the independent predictors of PP.Abbreviations – PP: polypharmacy

At any age PP was more common in HIV patients (fig. 4a). Drivers for higher PP risk were HIV duration with progressive OR increase per increment of HIV duration category and age above 75 years (fig. 4b).

A second model restricted to HIV patients only (data non-presented in fig. 4), while confirming the same predictors of the previous model, failed to identify any association between traditional HIV variables and PP. In particular: current CD4/CD8 OR = 0.83 (95% CI:0.44–1.52, *p* = 0.54), CD4 nadir OR = 1 (95% CI:1–1, *p* = 0.73), HIV RNA undetectability OR = 1.75 (95% CI:0.91–3.48, *p* = 0.1) and residual ARV exposure duration OR = 1.03 (95% CI:0.93–1.13, *p* = 0.61). Significant predictors identified in the original model were confirmed.

## Discussion

Our findings indicate that MM and PP in HIV-positive individuals are both related to longer duration of HIV-infection rather than older age per se.

People aging with HIV for more than 20 years are almost three times as likely to have MM than those infected for a shorter period.

It can be noticed that HIV positive patients belonging to the GEPPO cohort who have been living with HIV for more than 20 years were all exposed to the first generation of ART. These are “silver champions”, since they represent the best survivors of their generation and warrant as much attention as geriatric medicine has paid to centenarians, possibly identifying protective factors for NCDs.

These individuals have been exposed for years to detectable VL in the pre-ART era and have received highly toxic ART, both of them representing a permanent risk of NCDs.

However, it must be acknowledged that this phenomenon may change in future years. The START trial, a large randomized clinical trial conducted in 35 countries enrolling over 4500 HIV+ ART-naïve subjects randomized to immediate (CD4 ≥ 500/μL) or deferred ART initiation (CD4 < 350/μL) demonstrated that immediate ART reduced incidence of NCDs, pointing to the role of long-term immune activation and inflammation. In the contemporary setting of immediate access to new-generation ART, it can be hypothesised that the prevalence of NCDs and MM will be reduced in the years to come [23].

The prevalence of comorbidities was different in the HIV-positive patients and controls. The higher prevalence of DLM, CKD and T2DM has been widely described [12], and can at least partially be attributed to the metabolic toxicities of ART [24–26]. COPD was more prevalent in the HIV-negative individuals. This is somewhat surprising considering the higher proportion of smokers among HIV positive patients (26% vs 14%), and it suggests that infectious diseases physicians are less likely to use spirometry to screen HIV positive patients. The European AIDS Clinical Society (EACS) guidelines have only recently introduced COPD as a co-morbid condition that should be screened for [21]. There was no difference in the prevalence of CVD or HTN as has been observed in other cohorts [27], possibly due to strategies used to reduce CV risk factors, the increasing use of lipid-friendly ART agents, and reduction of immunodeficiency state.

Most of the participants of the GEPPO cohort have MM (59%), this appears to be the norm in HIV-infected geriatric patients [9]. We still need research to investigate the multifactorial nature of MM and the impact of this condition on quality of life, functional status impairment, health service use, and mortality. This will help healthcare services to address the unmet needs of PLWH with MM.

HIV, proportionally with its duration is a risk factor for PP. HIV positive patients have been visiting physicians since a young age and PP may be the result of the “medicalisation” of early diagnosis of NCDs.

MM and PP were both more prevalent in the HIV-positive group, respectively 64% and 37%. The increased burden of PP in this HIV cohort is striking, particularly in the light our very restrictive definition of PP (the chronic use of five or more drugs, excluding ART). The greater the number of medicines patient takes, the greater the risk of adverse effects, and the greater the risk of drug–drug interactions, leading to poor health outcomes, hospitalisations and mortality [28]. This is a dilemma for prescribers, who try to keep the number of medicines to a minimum while ensuring that patients receive what evidence-based guidelines advocate as being in their best interest [29].

Apparently, ID physicians and geriatricians use different drugs to treat the same comorbidities. An important difference regards the use of statins. Studies have underlined the need to increase statin prescription in HIV-positive patients due to increased cardiovascular risk. However, the use of statins in the elderly is a concern in the context of sarcopenia and fall risk [30]. Possibly geriatricians more than ID physicians are more concerned of this issue and this may be reflected by the fewer statin prescriptions received by the HIV negative controls in the GEPPO cohort.

Benzodiazepines were prescribed more frequently in the HIV negative than for the HIV positive patients. This may reflect the aversion of former intravenous drug users to use psychoactive drugs that may induce dependence.

With regard to gender, the prevalence and risk of MM (but not PP) was higher among males.

As expected males had increased risk of comorbidities but this was not the same for PP in the Italian national health system context were drugs for NCDs are provided for free in geriatric patients. The risks of MM and PP were different in the subjects aged more or less than 75 years. Apparently, the “younger-old” and “old” geriatric categories cover two subsets of elderly people with different risk profiles, and this must also be considered in HIV-positive patients.

Our data claim for a tailored approach to NCDs, and highlight the development of drug de-prescription strategies in the management of PP. Although de-prescribing is relatively new in HIV medicine, the use of the Beers criteria [31], IPET (Improving Prescribing in the Elderly Tool) [32] and STOPP-START criteria [33, 34] to adjust therapy and reduce potentially inappropriate drugs is well established in geriatric practice, and should be extended to the HIV setting [35].The benefit of de-prescribing in HIV positive geriatric patients has never been evaluated so far.

The GEPPO cohort includes HIV-positive patients aged ≥65 attending ten HIV clinics across Italy (who, taken together, makes a quite significant absolute number of elderly people living with HIV) and a group of age and gender-matched HIV-negative controls attending a single geriatric clinic. People of this age frequently visit geriatric clinics because of age-related comorbidities. Therefore, our cohort provides a new opportunity to compare HIV-infected patients with HIV-negative controls better representative of the general population than subjects attending centres for sexually transmitted diseases or intravenous drug user facilities used in previous studies.

This study has a number of limitations. Some of these are intrinsic to cross-sectional nature of observational studies, which cannot reveal any causative association between variables. The prevalence of comorbidities, although standardized in cohort studies may overestimate disease condition. This is the case of DLM, where use of statins is used as diagnostic criteria. We found no significant difference of HIV-related variables other than the duration of HIV infection to be associated with the risk of MM or PP. For this reason, ART exposure was not considered as a covariate, also because it requires properly designed clinically study. Cumulative smoke pack year was not addressed, because not routinely collected in all the clinics. A major limitation of this study was the lack of information on geriatric syndrome, including frailty and falls. Geriatric syndromes, better than NCDs capture the healthy aging outcome that all geriatric studies should address (ref). This information will be available in future studies of GEPPO cohort.

To the best of our knowledge, the GEPPO is the first geriatric cohort of HIV-positive patients that may contribute to identify unmet clinical and research needs in terms of comorbidities and their implications for PP. This model highlights the need for evidence-based screening and monitoring protocols to ensure high-quality care.

## Conclusions

The findings of this study show MM and PP burden in geriatric HIV positive patients are related to longer duration of HIV-infection rather than older age per se.
